# Supplementary material for: The dual role of amyloid-β-sheet sequences in the cell surface properties of FLO11-encoded flocculins in Saccharomyces cerevisiae
Source: eLife. 2021 Sep 1;10:e68592. doi: 10.7554/eLife.68592 (PMC8457840; doi:10.7554/eLife.68592)
Supplement: Supplementary file 1. [file elife-68592-supp1.docx]

**Supplementary File 1.** TANGO software analysis of β-aggregation motifs in Flo1, Flo5, Flo9, Flo10 and Flo11 protein from *Saccharomyces cerevisiae* S288c strain

| **Flo1** | | | **Flo5** | | | | **Flo9** | | | | | | | | | | **Flo10** | | | | | | | | | | | | | **Flo11** | | | | |  |  |
| --- | --- | --- | --- | --- | --- | --- | --- | --- | --- | --- | --- | --- | --- | --- | --- | --- | --- | --- | --- | --- | --- | --- | --- | --- | --- | --- | --- | --- | --- | --- | --- | --- | --- | --- | --- | --- |
| AA  position | motif | Mean β aggregate (%) | AA  position | motif | Mean β-aggregate  (%) | | | AA  position | | motif | | | Mean β-aggregate  (%) | | | | | | AA position | | | motif | | | | Mean β-aggregate  (%) | | AA  position | | | | motif | | Mean β-aggregate  (%) | |  |
| 7-21 | YMFLAVFTLLALTSV | 82.2 | 9-22 | IFLVILAFLALINVA | 82 | | | 6-22 | | **Y**YCLLLAIVTLLGLTNVV | | | | 77.55 | | | | | 7-23 | | | YIFLTGLFLLSVANVAL | | | | | 81.7 | 5-16 | | | | | FLLAYLVLSLLF | 96 | |  |
| 308-312 | TVIVI | 87.9 | 207-215 | TVYMYAGYY | 30.5 | | | 118-122 | | IIAYW | | | | | 72 | | | | 1028-1035 | | | VVTVYSTW | | | | | 48.5 | 1033-1042 | | | | VTTVVSTTVV | | 75.8 | |  |
| 353-357 | TIIVI | 87.3 | 308-312 | TVIVI | 73.2 | | | 207-215 | | TVYMYAGFY | | | | | 50.9 | | | | 1115-1119 | | | VLISV | | | | | 47 | 1056-1061 | | | | ITTTFV | | 56 | |  |
| 398-402 | TIIVI | 87.3 | 353-357 | TVIVI | 87.8 | | | 308-312 | | TVIVI | | | | | 87.9 | | | | 1157-1168 | | | ISIFIASLLLAI | | | | | 89.8 | 1133-1144 | | | | TLVTTAVTTTVV | | 84.8 | |  |
| 443-447 | TIIVI | 87.3 | 398-402 | TVIVI | 87.8 | | | 353-357 | | TIIVI | | | | | 87.3 | | | |  | |  | | | | |  | | 1356-1362 | | | | | FMWLLLA | 85.3 | |  |
| 488-492 | TIIVI | 87.3 | 443-447 | TVIVI | 87.8 | | | 398-402 | | TIIVI | | | | | 87.3 | | | |  | |  | | | | |  | |  | | | | |  |  | |  |
| 533-537 | TIIVI | 87.2 | 488-492 | TVIVI | 87.8 | | | 443-447 | | TIIVI | | | | | 87.3 | | | |  | |  | | | | |  | |  | | | | |  |  | |  |
| 578-562 | TIIVI | 87.2 | 533-537 | TVIVI | 87.9 | | | 498-492 | | TIIVI | | | | | 87.3 | | | |  | |  | | | | |  | |  | | | | |  |  | |  |
| 623-627 | TIIVI | 87.2 | 578-582 | TVIVI | 87.9 | | | 533-537 | | TIIVI | | | | | 87.2 | | | |  | |  | | | | |  | |  | | | | |  |  | |  |
| 667-672 | TIIVI | 87.2 | 623-627 | TVIVI | 87.9 | | | 578-582 | | TIIVI | | | | | 87.2 | | | |  | |  | | | | |  | |  | | | | |  |  | |  |
| 713-717 | TIIVI | 87.2 | 783-788 | TLVTVT | 31.7 | | | 623-627 | | TIIVI | | | | | 87.2 | | | |  | |  | | | | |  | |  | | | | |  |  | |  |
| 758-762 | TVIVI | 87.8 | 802-811 | AIVSTATVTV | 45.2 | | | 668-672 | | TIIVI | | | | | 87.2 | | | |  | |  | | | | |  | |  | | | | |  |  | |  |
| 803-807 | TVIVI | 87.8 | 855-859 | TVVTI | 37.2 | | | 713-717 | | TIIVI | | | | | 87.2 | | | |  | |  | | | | |  | |  | | | | |  |  | |  |
| 848-852 | TVIVI | 87.8 | 906-911 | TLVTVT | 36.1 | | | 758-762 | | TVIVI | | | | | 87.8 | | | |  | |  | | | | |  | |  | | | | |  |  | |  |
| 893-897 | TVIVI | 87.8 | 1063-1074 | LSVFIASLLLAI | 87 | | | 803-807 | | TVIVI | | | | | 87.8 | | | |  | |  | | |  | | | |  | | | | |  |  | |  |
| 938-942 | TVIVI | 87.8 |  |  | |  | | | 848-852 | | TVIIV | | | | | 87.,7 | | |  | |  | | | |  | | |  | | | |  | |  | | |
| 983-987 | TVIVI | 87.8 |  |  | |  | | | 1021-1026 | | TLVTVT | | | | | 31.9 | | |  | |  | | | |  | | |  | | | |  | |  | | |
| 1028-1032 | TVIVI | 87.9 |  |  | |  | | | 1040-1049 | | AIVSTATVTV | | | | | 42.6 | | |  | |  | | | |  | | |  | | | |  | |  | | |
| 1073-1077 | TVIVV | 88.5 |  |  | |  | | | 1093-1097 | | TVVTI | | | | | 37.4 | | |  | |  | | | |  | | |  | | | |  | |  | | |
| 1234-1239 | TLVTVT | 31.7 |  |  | |  | | | 1144-1149 | | TLVTVT | | | | | 37.7 | | |  | |  | | | |  | | |  | | | |  | |  | | |
| 1254-1262 | IVSTATVTV | 45.9 |  |  | |  | | | 1175-1182 | | VVTVYSTW | | | | | 82.6 | | |  | |  | | | |  | | |  | | | |  | |  | | |
| 1299-1303 | TVVTI | 37.2 |  |  | |  | | | 1310-1321 | | LSVFIASLLLAI | | | | | 87 | | |  | |  | | | |  | | |  | | | |  | |  | | |
| 1350-1355 | TLVTVT | 36 |  |  | |  | | |  | | |  |  | | | | |  | |  | | |  | | | | | |  | |  | | |  |  |  |
| 1525-1536 | LSVFIASLLLAI | 87 |  |  | |  | | |  | | |  |  | | | | |  | |  | | |  | | | | | |  | |  | | |  |  |  |
