# Supplementary material for: The dual role of amyloid-β-sheet sequences in the cell surface properties of FLO11-encoded flocculins in Saccharomyces cerevisiae
Source: eLife. 2021 Sep 1;10:e68592. doi: 10.7554/eLife.68592 (PMC8457840; doi:10.7554/eLife.68592)
Supplement: Supplementary file 2. — (a) Search for intragenic repeats using EMBOSS ETANDEM software. (b) Search for β-aggregation-prone sequence in the different Flo11 proteins using TANGO software (http://tango.crg.es/). *β-Aggregation-prone sequences > 30% were searched using TANGO software (at http://tango.crg.es/) with default setting of pH, ionic strength, and temperature. Amyloid-core sequences are highlighted in yellow. [file elife-68592-supp2.docx]

**Supplementary File 2:** Identification of sequence repeats and beta-aggregation prone sequence in Flo11 proteins from different yeast strains

**a.** intragenic repeats using EMBOSS ETANDEM software

| **Gene**  **name** | | **ORF (bp)** | | **Repetition**  **length**  **(TR)** | **Score** | **count** | **Repetition start**  **(nt. seq.)** | **Repetition stop**  **(nt. seq.)** | **Repetition conservation (%)** | **Repeated sequence (consensus)** |
| --- | --- | --- | --- | --- | --- | --- | --- | --- | --- | --- |
| *FLO11*^L69^ | 5166 | | 63 | | 323 | 30 | 658 | 2547 | 60.2 | acttcatctaccgctactactgcaaccacttctactactgcaaccacttctactactgcaaca |
|  |  |  | 45 | | 95 | 4 | 2646 | 2825 | 88.9 | accagctccaactccatccagctctactactgaaagctcttctgc |
|  |  |  | 45 | | 71 | 4 | 2958 | 3137 | 82.2 | atccagctctaccactgaaagctcttctgctccagtatcaacccc |
|  |  |  | 12 | | 41 | 8 | 2836 | 2955 | 73.3 | agctctactgctcca |
| *FLO11*^BY^ | 4104 | | 63 | | 304 | 24 | 688 | 2388 | 60.8 | tctactacagcaaccacttcaaccaccgcaactactgcaaccacttctactactgaaaccact |
|  |  |  | 33 | | 55 | 8 | 2832 | 3095 | 66.7 | ctctgcatgaacaaccactaccactacaactac |
|  |  |  | 45 | | 48 | 3 | 2429 | 2563 | 84.4 | caaccccatcaagctctagcactgaaagctcttctgctccagtat |
|  |  |  | 72 | | 24 | 4 | 3099 | 3386 | 66.7 | aactacagttttctccccaaacactgttactactacggtttcttctacaactacaactggtgcagacactac |
| *FLO11*^Σ^ | 3633 | | 81 | | 438 | 13 | 767 | 1819 | 74.6 | caaccagctctaccactgaaagctcttctgctccagctccaactccaaccagctctaccactgaaagctcttctgctccag |
|  |  |  | 45 | | 94 | 5 | 1923 | 2147 | 80.9 | cactgaaagctcttctgctccagtaccaactccatccagctctag |
|  |  |  | 45 | | 46 | 3 | 2158 | 2292 | 83.7 | ccagtaccaactccatccagctctagcactgaaagctcctctgct |
|  |  |  | 45 | | 29 | 2 | 335 | 424 | 91.1 | gttgcgacgaaaatacctatttgattgacaacccaactgatttca |
| *FLO11*^133d^ | 4890 | | 81 | | 1708 | 49 | 827 | 4795 | 72.5 | cttcttctgctccagttacttcttctactactgaatcttcttctgctccagctcctactccttcttcttctactactgaat |

*FLO11^69^* = *FLO11* gene from L69 strain; *FLO11^BY^* = FLO11 gene from BY4741 strain; *FLO11^Σ^* = FLO11 gene from Σ1278b; *FLO11^133d^ =* *FLO11* gene from 133d flor strain

**Supplementary file**

**2b.** β-aggregation prone sequence in the different Flo11 proteins using TANGO software (<http://tango.crg.es/> )

| **Protein**  (strain origin) | **Amino-acid position** | **Motif** | **Mean β aggregation (%)** |
| --- | --- | --- | --- |
| **Flo11p^69^**  (L69) | 5...16 | FLLAYLVLSLLF | 96 |
|  | 1178...1187 | VTTVVSTTVV | 75.7 |
|  | 1201...1206 | ITTTFV | 55.8 |
|  | 1285...1294 | VTTVVSTTVV | 75.7 |
|  | 1308...1313 | ITTTFV | 55.8 |
|  | 1392...1401 | VTTVVSTTVV | 75.7 |
|  | 1415...1420 | ITTTFV | 55.8 |
|  | 1494...1503 | VTTAVTTTVV | 59.4 |
|  | 1710...1716 | FMWLLLA | 85.3 |
| **Flo11p^BY^**  (BY4741) | 5...16 | FLLAYLVLSLLF | 96 |
|  | 1033...1042 | VTTVVSTTVV | 75.8 |
|  | 1056...1061 | ITTTFV | 56 |
|  | 1133...1144 | TLVTTAVTTTVV | 84.8 |
|  | 1356...1362 | FMWLLLA | 85.3 |
| **Flo11p^Σ^**  (Σ1278b) | 5...20 | FAYLVLSLLFYSAL | 83 |
|  | 881...890 | VTTVVSTTVV | 75.6 |
|  | 904...909 | ITTTFV | 55.4 |
|  | 983...992 | VTTAVTTTVV | 59 |
|  | 1199...1205 | FMWLLLA | 85.3 |
| **Flo11p^133d^**  (133d) | 5...16 | FLLAYLVLSLLF | 96 |
|  | 1301...1311 | VTTVVSTTVVT | 70.9 |
|  | 1324...1330 | ITTTFVT | 50.7 |
|  | 1403..1412 | VTTAVTTTVV | 59.1 |
|  | 1619...1625 | FMWLLLA | 85.3 |

*Beta-aggregation prone sequences > 30% were searched using TANGO software (at <http://tango.crg.es/>) with default setting of pH, ionic strength and temperature. Amyloid-core sequences are highlighted in yellow.
