# Supplementary material for: The dual role of amyloid-β-sheet sequences in the cell surface properties of FLO11-encoded flocculins in Saccharomyces cerevisiae
Source: eLife. 2021 Sep 1;10:e68592. doi: 10.7554/eLife.68592 (PMC8457840; doi:10.7554/eLife.68592)
Supplement: Supplementary file 4. [file elife-68592-supp4.docx]

**Supplementary file 4.** Yeast strains used or constructed in this study.

| **Strain** | **Genotype/Remarks** | | **Source** |
| --- | --- | --- | --- |
| L69 | *Saccharomyces cerevisiae* prototrophic diploid. | Lallemand Inc. | |
| L69*flo11∆* | L69 *flo11::KanMX4*. | This study | |
| L69*flo11∆-Nter* | L69 diploid strain expressing *FLO11* deleted of its N-terminus domain. | This study | |
| L69*flo11∆-RR1* | L69 diploid strain expressing *FLO11* deleted of the intragenic RR1. | This study | |
| L69*flo11∆-RR2* | L69 diploid strain expressing *FLO11* deleted of the RR2 intragenic domain | This study | |
| L69*flo11∆-Cter* | L69 diploid strain expressing *FLO11* deleted of its C-terminal domain | This study | |
| BY4741 | MAT**a** *his3Δ1 leu2Δ0* *met15Δ0* *ura3Δ0.* | Euroscarf | |
| BYflo11∆ | BY4741 *flo11::KanMX4*. | YKO Open biosystem | |
| Σ1278b | Prototophic diploid strain | Lab source | |
| YSWT3α | MATα can1Δ::Ste2pr-spHIS5 lyp1Δ::Ste3pr-LEU2 his3::hisG leu2Δ0 ura3Δ0. Derived from Σ1278b | C.Boone (Univ. Toronto) | |
| Σflo11Δ | YSWT3α flo11 ::kanMX4 | C.Boone (Univ. Toronto) | |
| BYflo11∆ *FLO11*^BY+^ | BY4741 *flo11::KanMX4* transformed with pYES *FLO11*^BY^. | This study | |
| BYflo11∆ *FLO11*^L69+^ | BY4741 *flo11::KanMX4* transformed with pYES-*FLO11*^L69^ | This study | |
| BYflo11∆*FLO11*^BY^[RR2]^L69+^ | BY4741 *flo11::KanMX4* haploid strain transformed with pYES-*FLO11[RR2]*^L69^ | This study | |
| Σflo11Δ *FLO11*^BY+^ | YSWT3α *flo11 ::kanMX4* strain transformed with pYES-*FLO11*^BY^ | This study | |
| Σflo11Δ *FLO11*^L69+^ | YSWT3α *flo11 ::kanMX4* strain transformed with pYES-*FLO11^L69^* p | This study | |
| BY4741+pFLO8 | BY4741 transformed with pGP564_FLO8 vector. | This study | |
| A9 | *MAT***a**/*MATHO/HO* | M. Budroni (Univ di Sassari, Italy) | |
