# Supplementary material for: The dual role of amyloid-β-sheet sequences in the cell surface properties of FLO11-encoded flocculins in Saccharomyces cerevisiae
Source: eLife. 2021 Sep 1;10:e68592. doi: 10.7554/eLife.68592 (PMC8457840; doi:10.7554/eLife.68592)
Supplement: Supplementary file 5. [file elife-68592-supp5.docx]

**Supplementary File 5:** Plasmids constructed in this work

| **Plasmids** | **description** | **Source** | |  |
| --- | --- | --- | --- | --- |
| pYES2.1 TOPO TA | E.coli /yeast shuttle vector with His Tag (6x), V5 Epitope Tag, Amp^R^, *GAL1* promotor, *URA3* marker. | | Life Technologies | |
| pYES-*FLO11*^L69^ | Cloning of *FLO11*^L69^ into pYES2.1 TOPO vector. | | This study | |
| pYES-*FLO11*^BY^ | Cloning of *FLO11*^BY^ into pYES2.1 TOPO vector. | | This study | |
| pYES-*FLO11^BY^-[RR2]*^L69^ | Cloning of *FLO11^BY^[RR2]*^L69^ chimeric gene in pYES2.1 | | This study | |
| pGP564_*FLO8* | pGP564 vector carrying GLE2, YER107W-A, FLO8, KAP123 and SWI4 | | Open Biosystem | |
