# Supplementary material for: The dual role of amyloid-β-sheet sequences in the cell surface properties of FLO11-encoded flocculins in Saccharomyces cerevisiae
Source: eLife. 2021 Sep 1;10:e68592. doi: 10.7554/eLife.68592 (PMC8457840; doi:10.7554/eLife.68592)
Supplement: Supplementary file 6. [file elife-68592-supp6.docx]

**Supplementary file 6.** Oligonucleotides used in this study

| **Oligonucleotides/gRNA sequence** | **Nucleic acid sequence (5’-3’)** | |
| --- | --- | --- |
| **Construction of Flo11 variant in L69 strain** | | |
| SAPI_RNAg1_L69_∆Nter | atccaaatcactattcatgtcaa | |
| SAPI_RNAg2_L69_∆Nter | aacttgacatgaatagtgatttg | |
| HF1_L69_∆Nter | aatgtccgtgttcaaattaaataaaaatttagggcagttttatttaccttaacaaatatgacatctagcacttccgagtcatctaccactacatcaactaccacttcagaatcatctacat | |
| HF2_L69_∆Nter | atgtagatgattctgaagtggtagttgatgtagtggtagatgactcggaagtgctagatgtcatatttgttaaggtaaataaaactgccctaaatttttatttaatttgaacacggacatt | |
| SAPI_RNAg1_L69_∆RR1 | atctcagtggtggagctggatac | |
| SAPI_RNAg2_L69_∆RR1 | aacgtatccagctccaccactga | |
| HF1_L69_∆RR1 | catctagcacttccgagtcatctaccactacatcaactaccacttcagaatcatctacatcaactccatccagctctactactgaaagctcttctgctccagtaccaaccccatcaagc | |
| HF2_L69_∆RR1 | gcttgatggggttggtactggagcagaagagctttcagtagtagagctggatggagttgatgtagatgattctgaagtggtagttgatgtagtggtagatgactcggaagtgctagatg | |
| SAPI_RNAg1_L69_∆RR2 | atctctactagtacaaaaccagg | |
| SAPI_RNAg2_L69_∆RR2 | aaccctggttttgtactagtaga | |
| HF1_L69_∆RR2 | cttcacctaccacacctgtaactacagttgtctcaaccaccgtcgttactactgagtattactctgccggtgaaactacctctggatgctctccaaagactgtcacaaccactgttcctt | |
| HF2_L69_∆RR2 | aaggaacagtggttgtgacagtctttggagagcatccagaggtagtttcaccggcagagtaatactcagtagtaacgacggtggttgagacaactgtagttacaggtgtggtaggtgaag | |
| SAPI_RNAg1_L69_∆Cter | atctccgctggtaagacgacaac | |
| SAPI_RNAg2_L69_∆Cter | aacgttgtcgtcttaccagcgga | |
| HF1_L69_∆Cter | tgagtattctactagtacaaaaccaggtggtgaaattacaactacatttgtcaccaaaaataaatacaattccaacatgttcgtttcttcattacgatgttttcattcttaaattaagat | |
| HF2_L69_∆Cter | atcttaatttaagaatgaaaacatcgtaatgaagaaacgaacatgttggaattgtatttatttttggtgacaaatgtagttgtaatttcaccacctggttttgtactagtagaatactca | |
| **Oligos for the construction of plasmids** | | |
| FLO11_TOPO_f | actatgcaaagaccatttctactcg | |
| FLO11_TOPO_r | gaatacaactggaagagcgagtagc | |
| FLO11_BY_2964_r | gcaaactgtagtagtaatagtggttgttga | |
| R269 inFLO11BY_f | tcaacaaccactattactactacagtttgc | |
| **Oligos used for the RT-qPCR** | | |
| FLO11_Nt_f | acccaactgatttcacagccac | |
| FLO11_Nt_r | agcttgcatattgagcggcac | |
| FLO11_Ct_f | gcaagcgcaggcgaaaacac | |
| FLO11_Ct_r | gattgaaatagggttggttgctgtg | |
| TAF10_f | atattccaggatcaggtcttccgtagc | |
| TAF10_r | gtagtcttctcattctgttgatgttgttgttg | |
| UBC6_f | gatacttggaatcctggctggtctgtctc | |
| UBC6_r | aaagggtcttctgtttcatcacctgtatttgc | |
| **Oligos use Immunofluorescence assay by addition of a 6-HIS tag at the C-terminal of Flo11 variants in L69 strain** | | |
| SAPI_ARNg1_HIStag_L69 | | atctaatgaagaaacgaacatgt |
| SAPI_ARNg2_HIStag_L69 | | aacacatgttcgtttcttcatta |
| HFf_histag_L69Ctermflo11Ct | | aaaaccaggtggtgaaattacaactacatttgtcaccaaaaacatcatcaccatcaccactaatgatacaattccaacatgttcgtttcttcattacgatgttttcattcttaaattaag |
| HFr_histag_L69Ctermflo11Ct | | cttaatttaagaatgaaaacatcgtaatgaagaaacgaacatgttggaattgtatcattagtggtgatggtgatgatgtttttggtgacaaatgtagttgtaatttcaccacctggtttt |
| PCR-HF_L69_fw1 | | gaatgttctgctgctacaaacg |
| PCR-HF_L69_rv1 | | ggaattgtatcattagtggtgatggtgatgatggaatacaactggaagagcgag |
| PCR-HF_L69_fw2 | | catcatcaccatcaccactaatgatacaattcctgatacaattccaacatgttcgtttct |
| PCR-HF_L69_rv2 | | gcctggtcgaagattattagttgt |
